# The International Phenological Garden network (1959 to 2021): its 131 gardens, cloned study species, data archiving, and future

**DOI:** 10.1007/s00484-021-02185-y

**Published:** 2021-09-07

**Authors:** Susanne S. Renner, Frank-M. Chmielewski

**Affiliations:** 1 Department of Biology, Washington University, Saint Louis, MO 63130 USA; 2 Division of Agricultural Climatology, Institute of Agricultural and Horticultural Sciences, Humboldt-University of Berlin, Berlin, Germany

**Keywords:** Plant phenology, Europe, Long-term data, Climate change, Monitoring

## Abstract

Collaborative networks that involve the compilation of observations from diverse sources can provide important data, but are difficult to maintain over long periods. The International Phenological Garden (IPG) network, begun in 1959 and still functioning 60 years later, has been no exception. Here we document its history, its monitored 23 species (initially all propagated by cloning), and the locations and years of data contribution of its 131 gardens, of which 63 from 19 countries contributed data in 2021. The decision to use clones, rather than multiple, locally adapted individuals, was based on the idea that this would “control” for genetic effects, and it affects the applicability of the data and duration of the network. We also describe the overlap among the IPG network, the Pan-European Phenology network (PEP725), and the phenological data offered by the German Weather Service. Sustainable data storage and accessibility, as well as the continued monitoring of all 23 species/clones, are under discussion at the moment, as is the fate of other phenological networks, despite a politically mandatory plant-based climate-change monitoring.

**Supplementary Information:**

The online version contains supplementary material available at 10.1007/s00484-021-02185-y.

## Introduction

Long-term ecological and environmental studies can contribute disproportionately to science and policy development (Hughes et al. 2017; Magnuson and Waide 2021), and there is a growing demand for long time series (i.e., data points indexed in time) especially in connection with climate change. The rapidly expanding capacity to detect correlations among variables, including with machine-learning approaches, underpins this interest in large and deep ecological data sets. In recognition of this, national and international agencies, beginning in the 1980s, set up long-term ecological and environmental study sites, such as the American Long-Term Ecological Research program (Magnuson and Waide 2021; https://lternet.edu, accessed 1 May 2021), the European LTER network (www.lter-europe.net, accessed 1 May 2021), and the International Long-Term Ecological Research network (https://lternet.edu/international/, accessed 1 May 2021). Surprisingly, phenological observations do not figure in these long-term research programs. Instead, data on plant phenology still tend to come from garden networks. The World’s oldest such network functioned from 1750 to 1752 and involved 18 estates distributed over the territory of Sweden (Linnaeus 1751; Ihne 1884; Schnelle 1955). The history of phenological gardens, that is, plantings of particular species for the purpose of monitoring their phenology, shows that most were founded from the 1950 onwards, with a peak in the 1980s, at least in Europe (Schnelle 1955; Ungersböck 2012).

**Fig. 1:**
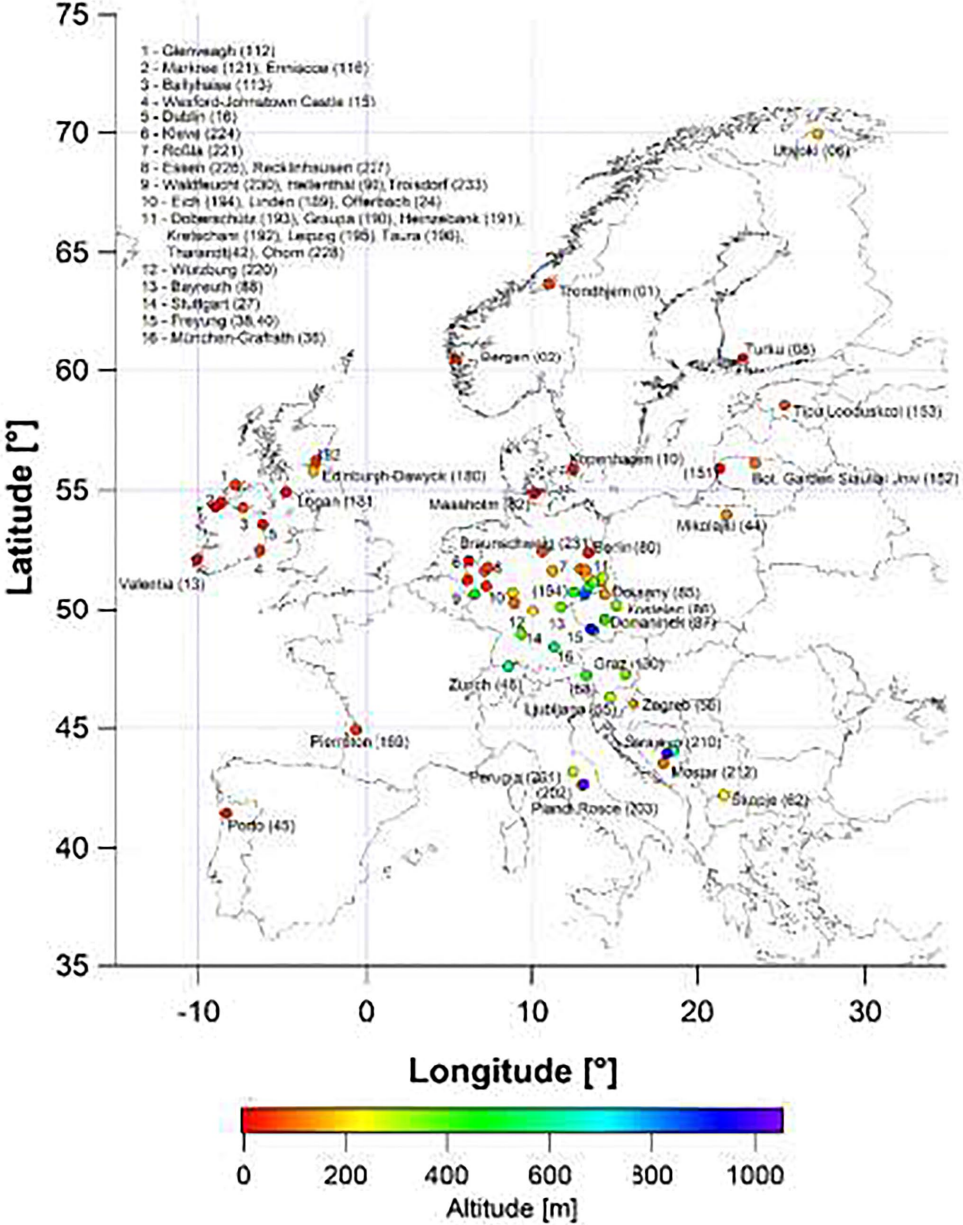
Map of the International Phenological Gardens active in 2021

Among the longest-running phenological networks are those of Japan, where phenological data have been gathered by the Japan Meteorological Agency since 1953 (Doi et al. 2021), and countries in Europe, where phenological monitoring by the German Weather Service (DWD) goes back to 1922 (Kasper et al. 2014), and the International Phenological Garden (IPG) network was established in 1959 (Schnelle and Volkert 1957, 1964, 1974; Chmielewski et al. 2013; http://ipg.hu-berlin.deaccessed 1 June 2021). The IPG network gathers and data-bases the dates of leaf/needle unfolding, “May shoots” of gymnosperms, flowering, mature fruits, autumn coloring, and leaf/needle fall for 23 species in “phenological gardens” throughout Europe (Fig. 1, Tables S1 and S2). A phenological garden as defined in this network consists of specific clones (representing the various species) propagated at a central location and distributed to every new garden wanting to join the network. Initially, each species was cloned via the rooting of cuttings from a “mother” tree individual. More recently, grafting has become the main method (see “Results and discussion” section). Over the past 60 years, phenological gardens have been established on the grounds of regular botanical gardens, forestry gardens, agricultural research institution, and meteorological institutions.

To establish the value of the IPG network, we here summarize its history from 1959 until 2021, tabulate the locations of all gardens and the periods during which each contributed data, and provide a list of the studied species, including the years over which each was monitored. We also explain why the IPG project focused on cloned plants rather than plants sourced from locally adapted populations, a decision that determines the applicability of the data and the future development of the network. We also summarize the type of research carried out with IPG data and highlight the utility of the data.

## Materials and methods

Author FMC has been responsible for directing and maintaining the IPG network since 1996. He has access to letters and notes of the IPG founders, Fritz Schnelle (1900–1990) and Erik Volkert (1907–1980), which are currently kept in the IPG archive of the Institute of Agricultural and Horticultural Sciences at the Humboldt-University of Berlin. For this study, FMC carried out archival research in Berlin and contacted former colleagues at the DWD, while author SSR interviewed key players in the European plant phenological community, including Helfried Scheifinger at the Central Institute for Meteorology and Geodynamics, Vienna, Austria, the current head of the Pan-European Phenological Network, Markus Ungersböck, the data manager of the Pan-European Phenological Network in Salzburg, and Annette Menzel, Chair of Ecoclimatology, Department of Life Science Systems, Technical University of Munich in Freising, Germany. We also went through all 48 issues of the former project publication series *Arboreta Phaenologica*, Mitteilungen der Arbeitsgemeinschaft Internationale Phänologische Gärten (Table S3) to find details about garden lay-out, plant sourcing, and plant propagation.

## Results and discussion

### History of the IPG project

The establishment of an international phenological observation program was decided at the first meeting of the Agrometeorological Commission of the World Meteorological Organization (WMO) in 1953 (Chmielewski et al. 2013). The realization of the idea was led by F. Schnelle and E. Volkert, who coordinated the network until 1973. Schnelle had studied Agricultural Sciences, and his dissertation dealt with the influence of weather and climate on the quality of wheat (Chmielewski 2007). From 1936 to 1945, Schnelle was responsible for Germany’s phenological network, and from 1949 until his retirement, he headed the Agrometeorological Department at the DWD, where he was again responsible for phenological monitoring, including the IPGs. Volkert had studied forestry, and from 1955 until his retirement, he was a professor of forestry at the University of Goettingen (Schnelle 1980). His special interest was the vegetative propagation of trees and shrubs. Together, Schnelle and Volkert proposed to the WMO that an international phenological network should be established and focus on monitoring widespread long-lived, woody plants of economic importance, that each member garden should be close to a weather station (which was not always realized), and that the methods of observations should be highly standardized (Schnelle and Volkert 1957, 1964). Schnelle saw the IPG network mainly as a source of standardized phenological observations and data on the growth of woody plants under different climatic and soil conditions (Baumgartner 1979).

**Fig. 2:**
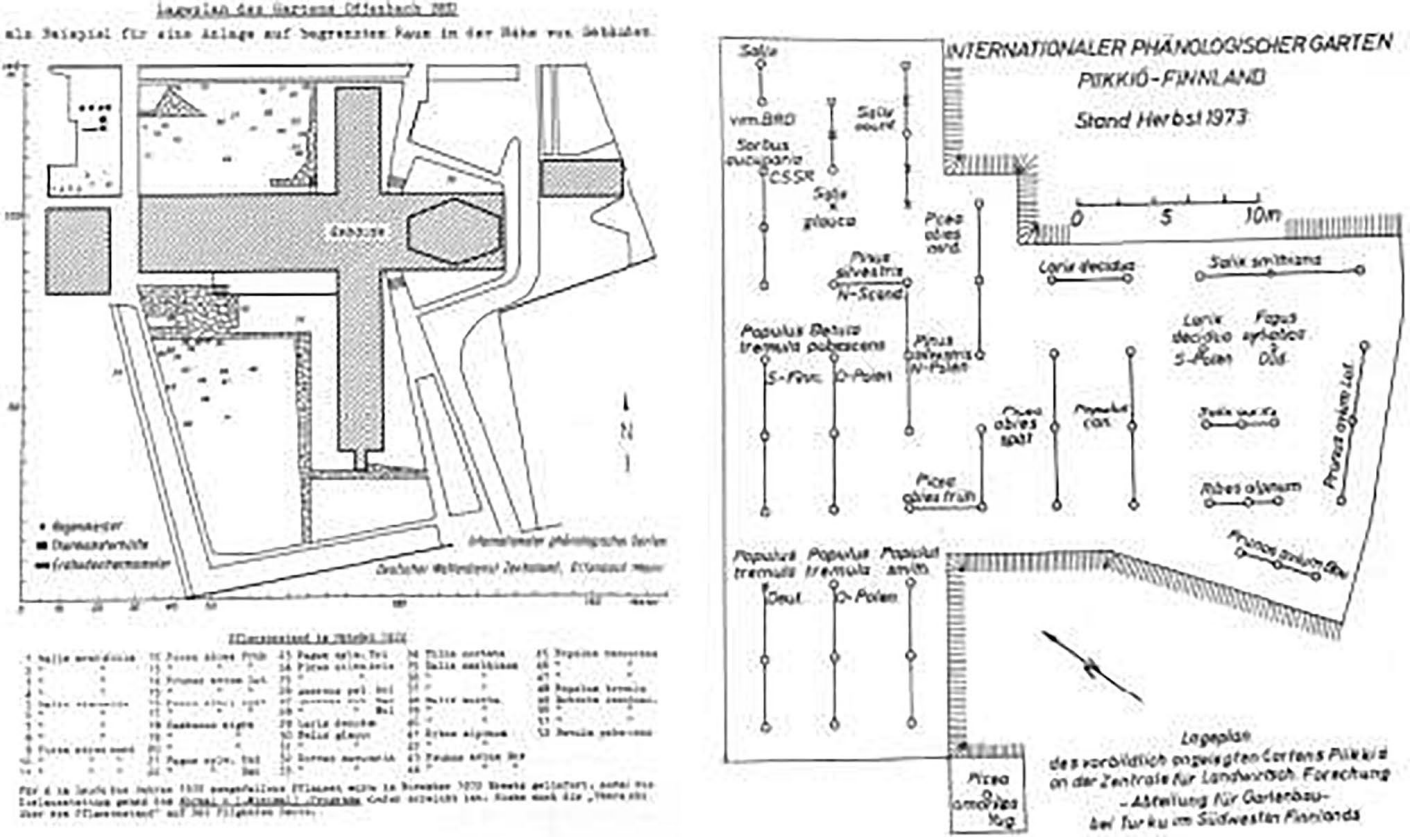
Layout of two International Phenological Gardens in the vicinity of buildings. On the left, garden number 24 in Offenbach, near buildings of the German Weather Service (drawing from *Arboreta Phaenologica* 17, 1972). On the right, garden number 8 in open terrain near Turku, Finland (drawing from *Arboreta Phaenologica* 18, 1973)

**Fig. 3:**
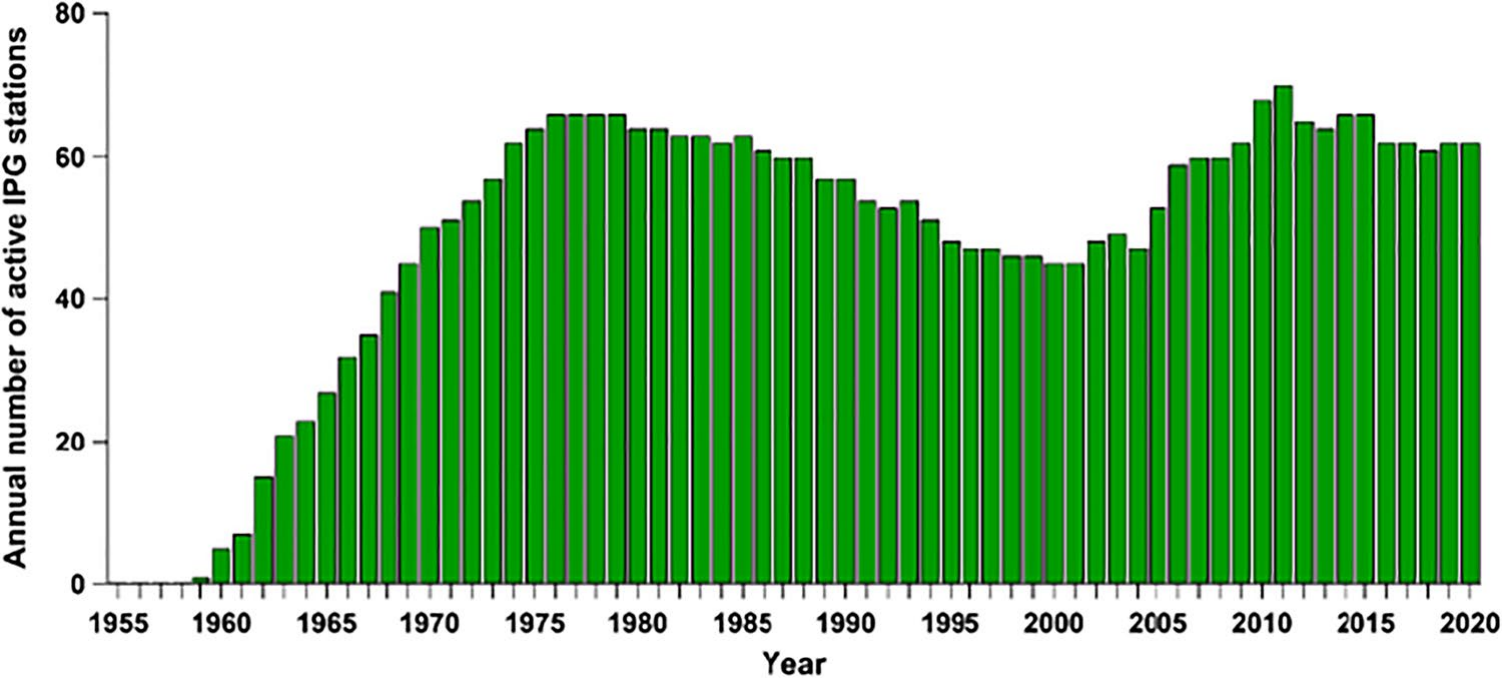
Number of International Phenological Gardens contributing annual data to the network between 1959 and 2020. This differs from a similar figure in Chmielewski et al. (2013; Fig. 8.1), which showed all established gardens, regardless of their reporting activity

The first garden was established in 1957 in Offenbach near Frankfurt (Fig. 2; Table S1), and the first observations, on five species (*Picea abies* early/late, *Populus canescens*, *Robinia pseudoacacia*, *Salix aurita*, *Salix smithiana*) began in 1959. In 1963, the network comprised 21 active gardens, and it continued to grow to 66 gardens between 1976 and 1979. The so-far highest number of simultaneously active IPGs, namely 77, was reached in 2011 (Fig. 3), and by 2021, as we write this, 131 IPGs have been initiated (Fig. 1, Table S1) of which 63 in 19 countries are currently active. Geographically, they span from ~ 63° N lat. in Norway to ~ 41° N lat. in Portugal, and from ~ 10° W long. in Ireland to ~ 25° E long. in Estonia (Fig. 1, Table S1).

From 1973 to 1977, the project was coordinated by Albert Baumgartner (1919–2008), chair of Bioclimatology and Applied Meteorology at the Technical University in Munich, with Schnelle continuing to collect data from the individual gardens and editing the *Arboreta Phaenologica* series (Table S3). Between 1978 and 1995, the German Weather Service took over the IPG management. Erika Freitag (1928–?), a researcher in the Agrometeorological Department of the German Weather Service, developed the initial electronic data base of the network (Freitag 1986, 1987; Freitag et al. 1993), and Helmut Lieth (1925–2015), an ecologist at the University of Osnabrück, served as the network’s scientific head. Since 1996, Frank-M. Chmielewski at the Institute of Agricultural and Horticultural Sciences at the Humboldt-University of Berlin has been coordinating and managing the network and has ensured its survival. He reorganized the plant reproduction and established a new non-commercial UNIX database (open source) with an online interface (http://ipg.hu-berlin.de), developed from the raw data he received from the German Weather Service as ASCII files.

### Species monitored and the decision to use cloned individuals

Schnelle and Volkert (1957) had originally proposed a network of plantings that should comprise two parts, one with genetically diverse individuals from widespread species, the other with cloned individuals. For the latter, they suggested 17 species (*Acer pseudoplatanus*, *Alnus rubra*, *Betula pendula*, *Fagus sylvatica*, *Larix decidua*, *Picea abies*, *Pinus sylvestris*, *Populus canescens*, *Populus tremula*, *Prunus avium*, *Quercus robur*, *Robinia pseudoacacia*, *Salix aurita*, *Salix caprea*, *Sambucus nigra*, *Sorbus aucuparia*, *Tilia cordata*), of which 14 made it into the final set used in the first gardens (Table S2; Schnelle and Volkert 1957). Between 1959 and 1968, *Betula pubescens*, *Ribes alpinum*, *Salix acutifolia*, *S. glauca*, *S. smithiana*, and *S. viminalis* were added to the program (Table S1). If a garden had sufficient space, further cloned provenances from Croatia, Denmark, France, Greece, Ireland, Italy, Poland, and Scandinavia were added in an “expanded set” (Table S2).

For most species, between two and eight clones were planted (Table S2), but for *Ribes alpinum*, *Robinia pseudoacacia*, *Sambucus nigra*, and *Tilia cordata*, a single clone was used (Table S2). The precise geographic origin of the “mother plants” has so far been documented for *Larix decidua*, *Prunus avium*, *Fagus sylvatica,* *Quercus petraea*, and *Q. robur* clones (our Table S2). The relevant information was retrieved from issue 11 of the annual report of the IPG network, “*Arboreta Phaenologica*, Mitteilungen der Arbeitsgemeinschaft Internationale Phänologische Gärten” (Volkert and Schnelle, 1968) and unpublished notes of Schnelle and Volkert that have passed into the possession of FMC.

**Fig. 4:**
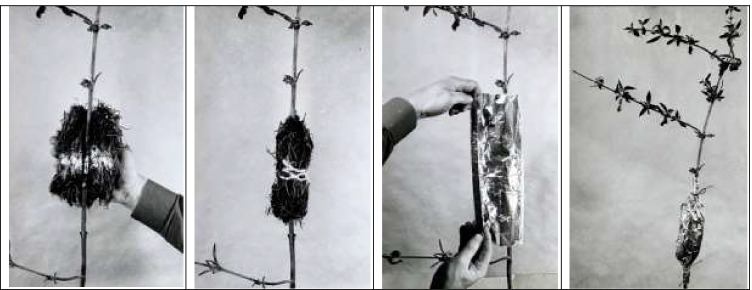
Propagation via aerial root formation used by Erik Volkert for the clonal propagation of *Fagus sylvatica*, *Quercus robur*, and *Tilia cordata* for the International Phenological Gardens, following a methodology developed by Herrmann (1967). Shown is the attachment of a padding consisting of humid peat with added plant growth hormone to a debarked piece of branch that is then covered with aluminum foil to prevent it from drying-out. Before the onset of winter, the treated branch is cut just below the padding and potted inside a greenhouse for overwintering and rooting. Branches that have developed roots are then planted outdoors in the spring

It is apparent from Schnelle’s obituary for Volkert (Schnelle 1980) that the use of cloned individuals for the IPG network was Schnelle’s idea, but that without Volkert’s expertise in the vegetative propagation of woody species, this undertaking could not have been realized. Schnelle and Volkert (1957, 1964) believed that one might “exclude genetic effects” if all gardens would use the same clones of each study species. They argued that the long-lived species selected for the phenological network should be propagated vegetatively so that all individuals go back to a single plant and thus have the same inner growth rhythm as their parent. For this, the vegetative propagation should be done by cuttings, not grafting, to guarantee that all plant parts from the root to the crown have the same genetic traits (Schnelle and Volkert 1957). To produce the required clones, a “parent” garden was needed in which plants would be propagated and then distributed to newly established gardens. Until 1964, this work was in the hands of H.-H. Heitmüller, a forester at the Institute for Forestry Genetics und Forest Tree Production Wächtersbach, near Frankfurt, today part of the German Research Institute of Forestry and Forest Products (https://www.thuenen.de/de/fg/, accessed 1 May 2021). Heitmüller achieved the propagation of most species via cuttings (earth rooting) and for three species (*Fagus sylvatica*, *Quercus robur*, and *Tilia cordata*) via areal rooting (Fig. 4, Table S2). Three species (*Pinus sylvestris*, *Prunus avium*, *Sorbus aucuparia*) could not be propagated by vegetative means and had to be produced by grafting (Volkert and Schnelle 1968) .

From 1964 to 1973, the Institute for Forest Plant Breeding in Escherode und Hannoversch Münden served as the parent garden and from 1973 to 1992, the Institute for Forestry Genetics und Forest Tree Production in Großhansdorf near Hamburg, today part of the Thünen Institut für Forstgenetik (https://www.thuenen.de/de/fg/, accessed 28 May 2021). Due to a lack of financial and human resources, plant propagation came to a standstill by 1982, although the parent garden in Großhansdorf was maintained until 1992.

When FMC took over running the IPG network in 1996, his first task was to find a new parent garden. In 1997, the Jordsand e.V. association took over this function, and young plants were moved from Großhansdorf to Ahrensburg, also near Hamburg. Mother trees that by then were 30 years old could not be moved, however. The garden in Ahrensburg turned out to be unable to propagate the original stock plants, and in 2001, they were therefore moved again, this time to a forestry test garden in Grafrath (Table S1), Bavaria, managed by the Bavarian State Institute for Forests and Forestry (https://www.lwf.bayern.de/service/lwf/007786/index.php, accessed 2 May 2021). It was decided to focus efforts on those 21 species for which mother plants were still available and to add three new taxa, *Corylus avellana*, *Forsythia suspensa*, and *Syringa x chinensis*, chosen because they were also in the Global Phenological Monitoring (GPM) program, which had just started (Chmielewski et al. 2013). The observation of *Syringa* was additionally motivated by the US lilac network, functioning since the 1950s (Schwartz et al. 2012). Because of the scarcity of vegetatively produced clones, which affected all species except *Salix* and the just-mentioned three new taxa, propagation by grafting became important by the beginning of the new millennium.

### Examples of the use of IPG network data in research

The use of cloned or grafted individuals in the IPG network since 1959 impacts the applicability of the data. The use of cloned plants clearly facilitates the comparison of observed phenological stages between gardens. Secondly, Schnelle and Volkert were interested in the plasticity of clones under different environmental conditions, and one such investigation was indeed carried out for *Picea abies* clones (Hanart-Rosch and Kleinschmit (1991). Other research used specific clones planted in the IPGs to develop phenological models. An example is a study of three Norway spruce clones from 23 gardens, one clone with an early timing of budburst from Germany and two with a late timing of budburst, originating from Germany and Norway (Olsson et al. 2017). Another study used clones of *Fagus sylvatica* and *Quercus robur* to test whether consistent patterns occur within clones planted along a gradient from colder climate to warmer climate IPGs (Wenden et al. 2020) . However, the small number of available clones in the authors’ view presented a problem. This was also the case for a study on the effects of light and temperature on bud burst in *Betula pubescens*, *Fagus sylvatica*, *Salix smithiana*, and *Tilia cordata*, whose authors concluded that “one specific tree clone for each species, [was used] and intra-specific variability in phenology was [thus] not considered, so the present findings need further validation before they can be generalized” Caffarra and Donnelly 2010, p. 720).

An alternative approach to overcoming the problem of few clones per species is to instead average across species. For example, the leaf-out and leaf-fall dates of four species, each presented by one or two clones in 51 gardens and monitored between 1969 and 1998, were averaged to document changes in the “mean” European growing season over that time (Chmielewski and Rötzer 2001, 2002; Rötzer and Chmielewski 2001). Yet another use of the clones was made by Linkosalo et al. (2019) who studied the transplanting success of valuable nursery trees to regions with different spring temperature. For this, they wanted to develop a thermal time model for *Tilia cordata* and used the precise phenological observations from the single clone of this species used in all IPGs. Clonally propagated IPG plants have also been used for measuring physiological characteristics, such as biogenic volatile organic compounds (van Meeningen et al. 2016) and may be useful in yet other ways in the future.

### Differences and overlap between the phenological databases maintained by the German Weather Service, the Pan-European PEP725 project, and the International Phenological Gardens

Germany’s first phenological monitoring program was set up by the Biological Institute for Agriculture and Forestry of the Weimar Republic in 1922 (Kasper et al. 2014). It ran until 1936, when it was taken over by the Meteorological Service of the Third Reich and further developed by Fritz Schnelle (Schnelle 1955). This phenological network is today operated by Germany’s DWD (Kasper et al. 2014). It includes 46 woody and herbaceous species (listed in Kaspar et al. 2014) of which 12 are also monitored in the IPG (highlighted in red in our Table S2). By 2014, about 1200 observers contributed to this network, the large majority of them on a voluntary basis. At some 60 sites, the observations are instead performed by observers paid by the DWD. The number of voluntary phenological observers is dwindling (Yuan et al. 2021), and the DWD webpages therefore have included calls for new volunteers as well as links to a citizen science phenology program. The phenology data are publicly available via the Climate Data Centre of the DWD (http://www.dwd.de/phaenologie, accessed 1 May 2021).

Inspired by the development of phenology in the 1990s and early 2000s, European researchers interested in phenology wrote an application for the European Cooperation in the field of Scientific and Technical Research (COST), which was funded as “Action 725: Establishing a European Phenological Data Platform for Climatological Applications (2005–2009).” Following the initial funding, which ended in 2009, a Pan-European Phenological database (PEP725; www.pep725.eu, accessed 1 May 2021) was set up by the Austrian Central Institute for Meteorology and Geodynamics, the Austrian Federal Ministry of Science and Research, and the Economic Interest Grouping of European National Meteorological Services (EUMETNET). The main aim of PEP725 is to promote and support phenological research by making available an annually updated pan-European database, providing open access to phenological data for science, research, and training (Kasper et al. 2014). The observed plants are mainly European wild species, except for a few cultivated fruit tree species. All species are monitored in the same way, following definitions of the phenological stages in the national networks.

By 2018, 32 European meteorological services and project partners from across Europe have supplied almost 12 million phenological records (since 1868 until the present) to the PEP725 database (Templ et al. 2018), and this data base also includes some (but not all) IPG observations from 1961 to 2000, in principle permitting a comparison between the phenology of naturally growing populations and the IPG clones, which are currently being identified as such (M. Ungersböck, Austrian Meteorological Service, Salzburg, personal communication, 28 May 2021).

By May 2021, the data available for downloads over the PEP725 web page end in 2016, but if one registers as a user, tailored data sets up to 2020 will be provided (M. Ungersböck, Austrian Meteorological Service, Salzburg, personal communication, 30 April 2021). The PEP725 database is currently funded by the Austrian Meteorological Service, but populating the database depends on individual gardens continuing to send data for incorporation into PEP725 in Salzburg. This is often a problem, and a recent update of a large study that had used the COST database to detect phenological trends in 542 plant species in 21 countries (125 628 time series) (Menzel et al. 2006) therefore ended up using data from the national meteorological phenological services of Germany, Switzerland, and Austria, instead of the PEP725 database, because “the national phenological databases are richer in sites, species and phenophases” (Menzel et al. 2020, p. 2600).

The IPG phenological data, which we described in the previous sections, differ from the phenological data gathered by the German Weather Service and by the Pan-European PEP725 project, in (i) the included taxa (12 IPG species also monitored by the German Weather Service are highlighted in Table S2), (ii) the length of the time series, and (iii) the reliance on clones and grafted material. Requests for data access have to be sent directly to the coordinator, currently author FMC.

### The IPG data gathered by 2021 and their safe-keeping for the future

Between 1959 and 2021, the IPG network collected more than 117 000 phenological observations for 23 species, which are stored in the IPG database and backed-up once a month on a separate server in the division of Agricultural Climatology at the Humboldt University of Berlin. Since 2010, observers are able to enter, view, and process their phenological observations and manage their garden’s data via an online interface of the database, accessible from the IPG homepage. For all gardens, the observed plant species are illustrated by photos. Besides the mean onset dates of the various phenological stages, annual data can also be interactively displayed on a map. Some IPGs provide information on climate parameters.

The IPG network is supported by two technical employees in the division of Agricultural Climatology, who are responsible for maintaining the database, processing data requests, and shipping plants from Berlin to new IPGs. Over the past 25 years (1996–2021), funding for the network has come mainly from the overhead of author FMC’s other research projects or from donations. How the network will be financed and maintained in the future is currently unresolved, similar to the situation at other phenological networks. Thus, the American National Phenology Network, begun in 2007, is also struggling to sort out long-term support (T. M. Crimmins, Director, USA National Phenology Network, email to numerous colleagues in the phenological community of 29 April 2021), and the Japanese network, too, suffers from a lack of staff and funding (Doi et al. 2021). Doi and colleagues suggested that in the future, volunteers might be recruited to make observations at each site, similar to Nature’s Notebook (http://usanpn.org/natures_notebook accessed 2 May 2021), iNaturalist in the USA (https://www.inaturalist.org/ accessed 2 May 2021), or the Naturgucker.de effort of the German Weather Service (https://www.naturgucker.info/vielfalt-studieren/naturguckermonitoring/phaenologie-mit-dwd/, accessed 28 May 2021).

For the IPG network, however, the main problem may not be volunteers to make the observations, but instead the continuation of the vegetative plant propagation, which continues to be the basis for the IPG network. It needs to be decided in which way grafting or cloning will be continued and whether the same approach should be used for all 23 species. For some, but not all, species, cuttings of the original clone for propagation are still available. Additionally, the IPG database will need to be updated and moved to a new server. Finding a future for the IPG network is crucial for safe-guarding the data collected since 1959. In the USA, one past phenological network already failed due to lack of funding (Doi et al. 2021). It would be a shame if the IPG network were to suffer the same fate.

## Supplementary Information

Below is the link to the electronic supplementary material.

Supplementary file1 (DOCX 31 KB)

Supplementary file2 (DOCX 18 KB)

Table S3 A list of the 48 issues of ‘*Arboreta Phaenologica*. Mitteilungen der Arbeitsgemeinschaft Internationale Phänologische Gärten’, published between 1959 and 2005, with a short summary of their contents. (DOCX 27 KB)
