# Supplementary material for: The International Phenological Garden network (1959 to 2021): its 131 gardens, cloned study species, data archiving, and future
Source: Int J Biometeorol. 2021 Sep 7;66(1):35–43. doi: 10.1007/s00484-021-02185-y (PMC8727390; doi:10.1007/s00484-021-02185-y)
Supplement: Supplementary file 1 — Supplementary file1 (DOCX 31 KB) [file 484_2021_2185_MOESM1_ESM.docx]

Table S1. The 131 International Phenological Gardens (IPGs) initiated between 1959 and 2021. Phenological gardens active by 2020 are shown in bold.

| **Country** | **IPG No.** | **Location** | **Latitude**  **N (°)** | **Longitude**  **E (°)** | **Altitude (m)** | **Observation period** |
| --- | --- | --- | --- | --- | --- | --- |
| Austria | 47 | Innsbruck-Stadt | 47.28 | 11.40 | 600 | 1973-1990 |
| Austria | 48 | Innsbruck-Rinn | 47.57 | 11.50 | 900 | 1968-1999 |
| Austria | 49 | Wien-Stad, Hohe-Warte | 48.25 | 16.37 | 202 | 1960-2015 |
| Austria | 50 | Wien-Obersiebenbrunn | 48.25 | 16.72 | 150 | 1960-1996 |
| **Austria** | **68** | **Salzburg** | **47.00** | **13.07** | **440** | **1985-2020** |
| Austria | 74 | Wien-Kobenzl | 48.27 | 16.30 | 450 | 1960-1965 |
| **Austria** | **130** | **Graz** | **47.05** | **15.41** | **355** | **2017-2020** |
| Belgium | 18 | Gent-Melle | 50.98 | 3.80 | 15 | 1963-1994 |
| Belgium | 19 | Bastogne-Michamps | 50.00 | 5.73 | 500 | 1972-1997 |
| Bosnia and Herzegovina | 58 | Sarajevo-Ivan Sedlo | 43.86 | 18.43 | 1000 | 1962-1991 |
| **Bosnia and Herzegovina** | **210** | **Sarajevo** | **43.86** | **18.43** | **630** | **2014-2020** |
| **Bosnia and Herzegovina** | **211** | **Sarajevo-Ivan Sedlo** | **43.75** | **18.04** | **967** | **2014-2020** |
| **Bosnia and Herzegovina** | **212** | **Mostar** | **43.33** | **17.80** | **99** | **2014-2020** |
| Bulgaria | 63 | Vidin-Bojuritza | 43.82 | 22.85 | 150 | 1969-1988 |
| Bulgaria | 64 | Velingrad-Yundola | 42.07 | 23.85 | 1490 | 1969-1988 |
| Bulgaria | 76 | Sofia | 42.68 | 23.33 | 564 | 1969-1970 |
| **Croatia** | **56** | **Zagreb-Krizevci** | **45.80** | **15.97** | **146** | **1962-2020** |
| Croatia | 59 | Dubrovnik-Trsteno | 42.72 | 17.98 | 64 | 1962-1985 |
| **Czech Republic** | **85** | **Prag-Doksany** | **50.47** | **14.17** | **158** | **2001-2020** |
| **Czech Republic** | **86** | **Prag-Kostelec** | **50.00** | **14.87** | **345** | **2002-2020** |
| **Czech Republic** | **87** | **Domanínek** | **49.32** | **14.17** | **457** | **2002-2020** |
| Denmark | 09 | København-Hørsholm | 55.87 | 12.50 | 40 | 1971-1985 |
| **Denmark** | **10** | **Kobhagen-Taastrup-Hojbakkegrad** | **55.67** | **12.30** | **30** | **1971-2020** |
| Denmark | 11 | Odense-Hofmansgave | 55.53 | 10.57 | 5 | 1976-1979 |
| Denmark | 12 | Aalborg-Tylstrup | 57.23 | 9.92 | 20 | 1972-1994 |
| **Estonia** | **153** | **Tipu Looduskool** | **58.36** | **25.05** | **83** | **2018-2020** |
| **Finland** | **06** | **Utsjoki-Kevo** | **69.75** | **27.00** | **180** | **1968-2020** |
| Finland | 07 | Oulu-Pelsonsuo | 64.52 | 26.45 | 115 | 1968-2008 |
| **Finland** | **08** | **Turku-Piikiö-Yltöinen** | **60.38** | **22.55** | **10** | **1965-2020** |
| France | 66 | Lieury-St. Pierre Sur Dives | 49.02 | -0.03 | 70 | 1978-1988 |
| France | 67 | Nancy-Champenoux | 48.75 | 6.35 | 250 | 1965-1965 |
| France | 165 | Arboretum National des Barres | 47.83 | 2.73 | 145 | 2006-2008 |
| **France** | **169** | **Pierroton** | **44.73** | **-0.78** | **58** | **2005-2020** |
| Germany | 20 | Münster | 51.97 | 7.63 | 60 | 1979-1990 |
| Germany | 21 | Hamburg-Schmalenbeck | 53.67 | 10.27 | 50 | 1961-1990 |
| Germany | 22 | Hamburg-Wulsdorf | 53.65 | 10.20 | 46 | 1970-2012 |
| Germany | 23 | Hannoversch Münden-Steinberg | 51.33 | 9.67 | 500 | 1965-1993 |
| **Germany** | **24** | **Offenbach** | **50.10** | **8.78** | **99** | **1959-2020** |
| Germany | 25 | Wiesbaden-Geisenheim | 49.98 | 7.97 | 109 | 1973-2013 |
| Germany | 26 | Trier | 49.75 | 6.67 | 265 | 1960-2000 |
| **Germany** | **27** | **Stuttgart-Hohenheim** | **48.72** | **9.22** | **380** | **1961-2020** |
| Germany | 28 | Stuttgart-Weilimdorf | 48.82 | 9.12 | 330 | 1968-1998 |
| Germany | 29 | Kaiserstuhl-Blankenhornsberg | 48.05 | 7.60 | 285 | 1972-2011 |
| Germany | 30 | Kaiserstuhl-Liliental | 48.07 | 7.68 | 265 | 1968-2009 |
| Germany | 31 | Freiburg-Stadt | 48.00 | 7.85 | 270 | 1970-2011 |
| Germany | 32 | Freiburg-Eschbach | 48.02 | 7.98 | 500 | 1976-2011 |
| Germany | 33 | Freiburg-Schauinsland | 47.92 | 7.90 | 1210 | 1970-2011 |
| Germany | 34 | Freiburg-Feldberg | 47.87 | 8.00 | 1370 | 1971-2010 |
| Germany | 35 | Donaueschingen | 47.95 | 8.52 | 680 | 1973-2011 |
| **Germany** | **36** | **München-Grafrath** | **48.18** | **11.17** | **540** | **1963-2020** |
| Germany | 37 | Freising-Weihenstephan | 48.40 | 11.73 | 460 | 1966-1992 |
| **Germany** | **38** | **Freyung-Schönbrunn** | **48.80** | **13.52** | **737** | **1974-2020** |
| Germany | 39 | Freyung-Klingenbrunn | 48.92 | 13.32 | 756 | 1974-2007 |
| **Germany** | **40** | **Freyung-Waldhäuser** | **48.93** | **13.33** | **956** | **1974-2020** |
| Germany | 41 | Freyung-Lusen | 48.93 | 13.52 | 1370 | 1976-2005 |
| **Germany** | **42** | **Tharandt-Hartha** | **50.98** | **13.54** | **360** | **1962-2020** |
| Germany | 73 | Quedlingburg | 51.95 | 11.13 | 123 | 1966-1968 |
| Germany | 77 | Hamburg-Quickborn | 53.73 | 9.88 | 13 | 1988-2015 |
| Germany | 78 | Berchtesgaden-Kühroint | 47.57 | 12.95 | 1430 | 1993-2003 |
| Germany | 79 | Berchtesgaden-Schapach | 47.58 | 12.97 | 950 | 1993-2003 |
| **Germany** | **80** | **Berlin-Thyrow** | **52.20** | **13.20** | **42** | **1999-2020** |
| Germany | 81 | Schleswig | 54.53 | 9.55 | 36 | 2002-2014 |
| **Germany** | **82** | **Maasholm** | **54.68** | **10** | **10** | **2006-2020** |
| Germany | 83 | Deuselbach | 49.67 | 7.05 | 480 | 1997-2003 |
| **Germany** | **88** | **Bayreuth** | **49.94** | **11.57** | **360** | **2006-2020** |
| **Germany** | **90** | **Hellenthal (Eifel)** | **50.48** | **6.43** | **470** | **2012-2020** |
| **Germany** | **189** | **Linden** | **50.53** | **8.68** | **172** | **2004-2020** |
| **Germany** | **190** | **Graupa** | **51.00** | **13.92** | **180** | **2005-2020** |
| **Germany** | **191** | **Heinzebank** | **50.68** | **13.12** | **610** | **2005-2020** |
| **Germany** | **192** | **Kretscham-Rothensehma** | **50.45** | **12.98** | **850** | **2005-2020** |
| **Germany** | **193** | **Doberschütz** | **51.53** | **12.7** | **99** | **2006-2020** |
| **Germany** | **194** | **Eich** | **50.55** | **12.33** | **444** | **2006-2020** |
| **Germany** | **195** | **Leipzig** | **51.20** | **13.13** | **198** | **2006-2020** |
| **Germany** | **196** | **Taura** | **51.47** | **13.02** | **124** | **2006-2020** |
| **Germany** | **220** | **Botanical Garden Univ. Würzburg** | **49.75** | **9.92** | **200** | **2012-2020** |
| **Germany** | **221** | **Roßla** | **51.45** | **11.07** | **156** | **2011-2020** |
| **Germany** | **224** | **Kleve** | **51.87** | **6.07** | **9** | **2013-2020** |
| Germany | 225 | Greifswalder Oie | 54.23 | 13.92 | 3 | 2011-2015 |
| **Germany** | **226** | **Essen** | **51.45** | **7.02** | **90** | **2010-2020** |
| **Germany** | **227** | **Recklinghausen** | **51.58** | **7.22** | **56** | **2010-2020** |
| **Germany** | **228** | **Ohorn** | **51.17** | **14.05** | **305** | **2012-2020** |
| Germany | 229 | Neschwitz | 51.27 | 14.35 | 155 | 2012-2012 |
| **Germany** | **230** | **Waldfeucht** | **51.07** | **6.0** | **32** | **2012-2020** |
| **Germany** | **231** | **Braunschweig** | **52.25** | **10.45** | **81** | **2013-2020** |
| Germany | 232 | Seniorenhaus Berghof | 50.02 | 6.28 | 420 | 2016-2017 |
| **Germany** | **233** | **Troisdorf** | **50.81** | **7.12** | **55** | **2019-2020** |
| Great Britain | 17 | London-Farnham-Headly Park | 51.08 | -0.89 | 84 | 1969-1981 |
| Great Britain | 71 | Oxford-Kennington | 51.67 | -1.30 | 60 | 1969-1970 |
| Great Britain | 72 | London-Farnham-Alice Holt | 51.20 | -0.78 | 80 | 1967-2015 |
| Great Britain | 173 | CEH Monks Wood | 52.42 | -0.25 | 43 | 2005-2006 |
| **Great Britain** | **180** | **RBG Edinburgh-Dawyck** | **55.60** | **-3.32** | **180** | **2010-2020** |
| **Great Britain** | **181** | **Logan** | **54.74** | **-4.96** | **30** | **2015-2020** |
| **Great Britain** | **182** | **Edinburgh-Inverleith** | **55.97** | **-3.21** | **50** | **2010-2020** |
| Greece | 65 | Saloniki-Serres-Lailia | 41.25 | 23.62 | 1550 | 1963-1987 |
| Hungary | 53 | Budapest-Gödöllö | 47.60 | 19.35 | 220 | 1974-1994 |
| Hungary | 54 | Debreczin-Püspökladany | 47.33 | 21.13 | 90 | 1973-1979 |
| **Ireland** | **13** | **Kerry-Valentia Observatory** | **51.93** | **-10.25** | **14** | **1966-2020** |
| Ireland | 14 | CO-Wexford-J.F. Kennedy Park | 52.33 | -6.63 | 80 | 1966-2011 |
| **Ireland** | **15** | **Wexford-Johnstwon Castle** | **52.30** | **-6.52** | **60** | **1967-2020** |
| **Ireland** | **16** | **Dublin National Botanical Gardens** | **53.38** | **-6.33** | **30** | **1966-2020** |
| **Ireland** | **112** | **Glenveagh National Park** | **55.03** | **-7.97** | **65** | **2008-2020** |
| **Ireland** | **113** | **Ballyhaise College** | **54.08** | **-7.52** | **77** | **2009-2020** |
| Ireland | 115 | Carton Estate | 53.39 | -6.57 | 60 | 2010-2011 |
| **Ireland** | **116** | **Enniscoe House** | **54.11** | **-9.16** | **3** | **2011-2020** |
| Ireland | 118 | Armagh Observatory | 54.35 | -6.65 | 64 | 2005-2011 |
| Ireland | 119 | Millstreet Country Park | 52.12 | -9.08 | 116 | 2010-2016 |
| **Ireland** | **121** | **Markree Castle** | **54.30** | **-8.82** | **40** | **2010-2020** |
| **Italy** | **201** | **Perugia** | **43.00** | **12.30** | **270** | **2009-2020** |
| **Italy** | **202** | **Rieti** | **42.42** | **12.82** | **380** | **2009-2020** |
| **Italy** | **203** | **Pian Di Rosce** | **42.67** | **12.93** | **1050** | **2009-2020** |
| **Lithuania** | **151** | **Botanical Garden of Klaipeda University** | **55.72** | **21.17** | **9** | **2007-2020** |
| **Lithuania** | **152** | **Botanical Garden Siauliai University** | **55.92** | **23.27** | **117** | **2007-2020** |
| **Macedonia** | **62** | **Skopje** | **42.00** | **21.43** | **240** | **1962-2020** |
| Netherlands | 69 | Wageningen | 51.98 | 5.67 | 25 | 1965-1978 |
| **Norway** | **01** | **Trondhjem-Stjørdal-Kvithamar** | **63.49** | **10.88** | **63** | **1964-2020** |
| **Norway** | **02** | **Bergen-Fana** | **60.27** | **5.35** | **50** | **1964-2020** |
| Norway | 03 | Oslo Ås-Vollebekk | 59.67 | 10.78 | 95 | 1963-1986 |
| Poland | 43 | Posnan-Kornik | 52.25 | 17.10 | 74 | 1970-1977 |
| **Poland** | **44** | **Mikolajki** | **53.78** | **21.58** | **127** | **1967-2020** |
| **Portugal** | **45** | **Porto** | **41.25** | **-8.50** | **30** | **1968-2020** |
| Portugal | 94 | Evora | 38.57 | -7.90 | 309 | 2004-2015 |
| Serbia | 57 | Sombor | 45.78 | 19.12 | 90 | 1975-1993 |
| Serbia | 60 | Bar | 42.08 | 19.08 | 5 | 1975-1993 |
| Serbia | 61 | Beograd-Smederevska-Palanka | 44.37 | 20.95 | 121 | 1974-1995 |
| Serbia | 75 | Beograd-Zeleno-Brdo | 44.78 | 20.53 | 245 | 1962-1972 |
| Slovakia | 51 | Slepcany-Mlynany | 48.33 | 18.37 | 180 | 1962-2011 |
| Slovakia | 52 | Zvolen-Kysihybel-Vulh | 48.45 | 18.93 | 540 | 1966-2012 |
| **Slovenia** | **55** | **Ljubljana** | **46.07** | **14.50** | **310** | **1962-2020** |
| Sweden | 04 | Lund-Svalöf-Ekebo | 55.97 | 13.33 | 50 | 1963-1979 |
| Sweden | 05 | Stockholm-Bogesund | 59.37 | 18.50 | 50 | 1970-1983 |
| **Switzerland** | **46** | **Zürich-Birmensdorf** | **47.36** | **8.44** | **600** | **1963-2020** |
