# Supplementary material for: The International Phenological Garden network (1959 to 2021): its 131 gardens, cloned study species, data archiving, and future
Source: Int J Biometeorol. 2021 Sep 7;66(1):35–43. doi: 10.1007/s00484-021-02185-y (PMC8727390; doi:10.1007/s00484-021-02185-y)
Supplement: Supplementary file 2 — Supplementary file2 (DOCX 18 KB) [file 484_2021_2185_MOESM2_ESM.docx]

**Table S2.** Plant species studied in the International Phenological Gardens between 1959 and 2021, with their years of first and last monitoring and type of propagation: ^1^cuttings (earth rooting), ^2^aerial root formation, ^3^grafts. The 23 species in the standard program are almost the same as those listed by Schnelle and Volkert (1964), except for the addition of *Corylus avellana*, *Forsythia suspensa*, and *Syringa x chinensis*. The 12 species also monitored by the German Weather Service (Kaspar et al., 2014) are highlighted in red. Provenance is given as precisely as possible.

| **Standard set**  **Species, provenance** | **Expanded set**  **Species, provenance** | **First**  **obs. year** | **Last**  **obs. year** |
| --- | --- | --- | --- |
| Gymnosperms |  |  |  |
| *Larix decidua*^1^  Germany, Schlitz, 50.68 N, 9.56 E, 245 m alt. |  | 1960 | 2020 |
|  | *Larix decidua*  Eastern France | 1972 | 2020 |
|  | *Larix decidua*  South Poland | 1972 | 2020 |
|  | *Larix decidua*  Czech Republic | 1972 | 2020 |
|  | *Larix decidua*  Northern Italy | 1973 | 2008 |
| *Picea abies*^1^ (early)  German*y* |  | 1959 | 2020 |
| *Picea abies* (late)  Germany |  | 1959 | 2020 |
| *Picea abies* (northern)  Southern Norway |  | 1962 | 2020 |
|  | *Picea abies* (late)  E-Poland | 1978 | 2020 |
|  | *Picea abies* (average)  E-Poland | 1982 | 1982 |
|  | *Picea abies*  E-Germany | 1977 | 2020 |
|  | *Picea abies*  E-France | 1977 | 2020 |
|  | *Picea abies*  N-Scandinavia | 1982 | 2009 |
|  | *Picea omorika*  Croatia | 1970 | 2020 |
| *Pinus sylvestris*^3^  N-Poland |  | 1967 | 2020 |
|  | *Pinus sylvestris*  E-France | 1970 | 2020 |
|  | *Pinus sylvestris*  N-Scandinavia | 1974 | 2020 |
| Angiosperms |  |  |  |
| *Betula pubescens*^1^  Germany |  | 1960 | 2020 |
|  | *Betula pendula*  N-Poland | 1972 | 2020 |
|  | *Betula pendula*  N-Scandinavia | 1977 | 2004 |
| *Corylus avellana*^1^  *Germany* |  | 2001 | 2020 |
| *Fagus sylvatica*^2^  Germany, Hardegsen, 51.65 N, 9.82 E, 191 m alt. |  | 1969 | 2020 |
| *Fagus sylvatica*  Germany, Düdelsheim, 50.29 N, 9.03 E, 125 m alt. |  | 1969 | 2020 |
| *Fagus sylvatica*  Germany, Trippstadt, 49.35 N, 7.77 E, 406 m alt. |  | 1969 | 2020 |
|  | *Fagus sylvatica*  Denmark, Hørsholm, 55.87 N, 12.50 E, 40 m alt. | 1970 | 2020 |
|  | *Fagus orientalis*  Balkans | 1970 | 2020 |
| *Forsythia suspensa fortunei*^1^  Germany |  | 2001 | 2020 |
| *Populus canescens*^1^  Germany |  | 1959 | 2020 |
| *Populus tremula*^1^  Germany |  | 1960 | 2020 |
|  | *Populus tremula*  E-Poland | 1971 | 2020 |
|  | *Populus tremula*  S-Finland | 1971 | 2020 |
|  | *Populus tremula*  Ireland | 1972 | 1998 |
|  | *Populus tremula*  Greece | 1974 | 1993 |
| *Prunus avium*^3^  Germany, Bovenden, 51.59 N, 9.49 E, 139 m alt. |  | 1962 | 2020 |
| *Prunus avium*  Germany, Lutter, 51.33 N, 10.12 E, 325 m alt. |  | 1962 | 2020 |
|  |  |  |  |
| *Quercus petraea*^2^  Germany, Zell, 50.03 N, 7.19 E, 100 m alt. |  | 1972 | 2013 |
|  | *Quercus petraea*  Croatia, Krstovi, 45.13 N, 16.41 E, 168 m alt. | 1973 | 2013 |
| *Quercus robur*^2^  Germany, Wolfgang, 50.13 N, 8.92 E, 108 m alt. |  | 1970 | 2020 |
| *Quercus robur*  Germany, Barlohe, 54.13 N, 9.61 E, 104 m alt. |  | 1970 | 2020 |
|  | *Quercus robur*  Croatia, Lipovljani, 45.40 N, 16.89 E, 168 m alt. | 1970 | 2020 |
| *Ribes alpinum*^1^  Austria |  | 1962 | 2020 |
| *Robinia pseudoacacia*^1^  USA |  | 1959 | 2020 |
| *Salix acutifolia*^1^  Germany |  | 1962 | 2020 |
| *Salix aurita*^1^  Germany |  | 1959 | 2020 |
| *Salix glauca*^1^  Greenland |  | 1963 | 2020 |
| *Salix smithiana*^1^  Germany |  | 1959 | 2020 |
| *Salix viminalis*^1^  Germany |  | 1968 | 2020 |
| *Sambucus nigra*^1^  Germany |  | 1961 | 2020 |
| *Sorbus aucuparia*^3^  Czech Republic |  | 1970 | 2020 |
|  | *Sorbus aucuparia*  N-Scandinavia | 1976 | 2017 |
| *Syringa x chinensis*^1^ cult. *Red Rothomagensis*  Germany |  | 2001 | 2020 |
| *Tilia cordata*^2^  Germany |  | 1970 | 2020 |
