# Supplementary material for: The International Phenological Garden network (1959 to 2021): its 131 gardens, cloned study species, data archiving, and future
Source: Int J Biometeorol. 2021 Sep 7;66(1):35–43. doi: 10.1007/s00484-021-02185-y (PMC8727390; doi:10.1007/s00484-021-02185-y)
Supplement: Supplementary file 3 — A list of the 48 issues of ‘Arboreta Phaenologica. Mitteilungen der Arbeitsgemeinschaft Internationale Phänologische Gärten’, published between 1959 and 2005, with a short summary of their contents. (DOCX 27 KB) [file 484_2021_2185_MOESM3_ESM.docx]

**ARBORETA PHAENOLOGICA**‘Mitteilungen der Arbeitsgemeinschaft Internationaler Phänologischer Gärten’

| **Issue #** | **Title** | **Year** | **Authors** | **# of pages** |
| --- | --- | --- | --- | --- |
|  | **Herausgeber: Dr. F. Schnelle Offenbach a. M. und Prof. Dr. E. Volkert Hann. Münden** |  |  |  |
| 1 | *Einrichtung der Internationalen Phänologischen Gärten* | 1958 | Schnelle, F.; Volkert, E. | 7+2 |
| 2 | *Anleitung für phänologische Beobachtungen in den Internationalen Gärten* | 1960 | Schnelle, F. | 10+2 |
| 3 | *Erläuterungen zur Beobachtung des phänologischen Termins „Blühbeginn“ bei der Salix smithiana* | 1966 | Schnelle, F.; Volkert, E. | 7+4 |
| 4 | *Erläuterungen zu Beobachtung des phänologischen Termins „Nadelentfaltung“ bei der Larix decidua* | 1966 | Schnelle, F.; Volkert, E. | 4 |
| 5 | *Erläuterungen zu Beobachtung des phänologischen Termins „Blattentfaltung“ bei Populus canescens* | 1966 | Schnelle, F.; Volkert, E. | 6 |
| 6 | *Erläuterungen zu Beobachtung des phänologischen Termins „Blattentfaltung“ bei der Betula pubescens* | 1966 | Schnelle, F.; Volkert, E. | 7 |
| 7 | *Erläuterungen zu Beobachtung des phänologischen Termins „Blattentfaltung“ bei der Populus tremula* | 1966 | Schnelle, F.; Volkert, E. | 5 |
| 8 | *Erläuterungen zur Beobachtung des phänologischen Termins „Nadelentfaltung“ bei Picea abies* | 1966 | Schnelle, F.; Volkert, E. | 9 |
| 9 | *Übersicht über die Temperatur- und Niederschlagsverhältnisse im Beobachtungsraum der Internationaler Phänologischer Gärten* | 1967 | Schnelle, F.; Volkert, E. | 6+7 |
| 10 | *Bericht über die Ergebnisse der phänologischen Beobachtungen bis 1965 an mehreren Internationaler Phänologischer Gärten in Europa* | 1967 | Schnelle, F.; Volkert, E. | 11+10 |
| 11 | *Stand und zukünftige Aspekte des Programms “Internationaler Phänologischer Gärten“* | 1968 | Schnelle, F.; Volkert, E. | 26+4 |
| 12 | *Bericht über die Ergebnisse der phänologischen Beobachtungen im Jahre 1966 an 29 Internationalen Phänologischer Gärten in Europa* | 1968 | Schnelle, F.; Volkert, E. | 4+5 |
| 13 | *Bericht über die Ergebnisse der phänologischen Beobachtungen im Jahre 1967 an 31 Internationalen Phänologischer Gärten in Europa* | 1968 | Schnelle, F.; Volkert, E. | 6+9 |
| 14 | *Gehölze zur phänologischen Beobachtung, Agrarmetrologische Forschungsstelle Weihenstephan des Deutschen Wetterdienstes* | 1970 | Schnelle, F.; Volkert, E. | 5+7 |
| 15 | *Bericht über die Ergebnisse der phänologischen Beobachtungen im Jahre 1969 an 41 Internationalen Phänologischer Gärten in Europa*  *Phenological Gardens: Lecture at UNESCO Seminar on Techniques of Measurements of Primary, Denmark* | 1971 | Schnelle, F.; Volkert, E.  Nielsen, C. | 1-6  6-10+8 |
| 16 | *Bericht über die Ergebnisse der phänologischen Beobachtungen im Jahre 1970 an  45 Internationalen Phänologischer Gärten in Europa* | 1971 | Schnelle, F.; Volkert, E. | 6+13 |
| 17 | *Lageplan des Gartens Offenbach, Deutschland* | 1972 | Schnelle, F.; Volkert, E. | 4+6 |
| 18 | *Internationaler Phänologischer Garten Pikkiö-Finnland: Stand Herbst 1973* | 1973 | Schnelle, F.; Volkert, E. | 4+7 |
|  | **Herausgeber: Prof. Dr. A. Baumgartner; Dr. F. Schnelle und Prof. Dr. E. Volkert** |  |  |  |
| 19 | *Internationaler Phänologischer Gärten: Beobachtungsergebnisse und neue Gärten ohne Beobachtungsergebnisse* | 1974 | Baumgartner, A.; Schnelle, F. | 5+10 |
| 20 | *Jahresunterschiede von Salix smithiana - Blühbeginn in verschiedenen Gebieten Europas* | 1975 | Baumgartner, A.; Schnelle, F. | 5+8 |
|  | **Herausgeber: Prof. Dr. A. Baumgartner; Dr. F. Schnelle, Prof. Dr. E. Volkert und Prof. Dr. K.-F. Schreiber** |  |  |  |
| 21 | *Phänologische Frühjahrsentwicklung in verschiedenen Gebieten Europas 1975* | 1976 | Baumgartner, A.; Schnelle, F. | 5+10 |
| 22 | *Vertikale Unterschiede der phänologischen Frühjahrsentwicklung 1976 in einigen Gebirgen Europas* | 1977 | Baumgartner, A.; Schnelle, F. | 5+10 |
|  | **Herausgeber: Prof. Dr. A. Baumgartner; Dr. F. Schnelle, Prof. Dr. E. Volkert und Dr. E. Freitag** |  |  |  |
| 23 | *Einige Frühjahrsphasen im Jahre 1977 im Vergleich zu 12jährigen Mittel- und Extremwerten der Periode 1966-1977* | 1978 | Baumgartner, A.; Schnelle, F. | 5+8 |
| 24 | *Stand und Fortentwicklung des Netzes der Internationalen Phänologischen Gärten* Bericht über die Ergebnisse der phänologischen Beobachtungen im Jahre 1978 an 66 Internationalen Phänologischer Gärten (IPG) in Europa. | 1979 | Baumgartner, A. Freitag, E. | 1-2 3-5+8 |
|  | **Herausgeber: Prof. Dr. A. Baumgartner; Dr. F. Schnelle und Dr. E. Freitag** |  |  |  |
| 25 | *Nachruf auf Professor Dr. Erik Volkert*  Bericht über die Ergebnisse der phänologischen Beobachtungen im Jahre 1979 an 66 Internationalen Phänologischer Gärten (IPG) in Europa. | 1980 | Schnelle, F  Freitag, E. | 1-2  3-6+8 |
| 26 | *Dr. Fritz Schnelle 80 Jahre*  Schnelles Verdienste um die Gründung und Erhaltung der Internationaler Phänologischer Gärten (IPG). Bemerkungen zu den phänologischen Beobachtungen an den Internationalen Phänologischen Gärten. | 1981 | Baumgartner, A. Freitag, E. | 1 2-3  4+8 |
| 27 | Jahressunterschiede der Zeitspanne (Tage) zwischen den phänologischen Phasen *Salix smithiana* (b) und *Picea abies* (M), Mittel aus *Picea abies* früh, spät und nördlich in den Jahren 1964 -1981. Bemerkungen zu den phänologischen Beobachtungen an den Internationalen Phänologischen Gärten im Jahre 1981. | 1982 | Schnelle, F.  Freitag, E. | 1-2  3+8 |
| 28 | *Beobachtungsergebnisse aus dem Internationalen Phänologischer Garten in Offenbach*  (1959-1983)  Bemerkungen zu den phänologischen Beobachtungen in den Internationalen Phänologischen Gärten (IPG) im Jahre 1982. | 1984 | Freitag, E. | 1-32 |
| 29 | *25 Jahre Internationaler Phänologischer Gärten* | 1985 | Schnelle, F. | 44 |
| 30 | Der phänologische Garten des Valentia-Observatoriums, Irland. Hinweise für die Betreuer der Internationalen Phänologischen Gärten (IPG). | 1986 | Murphy, E.; Keane, T. Freitag, E.; Polte, C. | 1-15 16-39 |
| 31 | *1. Sonderheft zum Internationalen Phänologie-Symposium an der Universität für Bodenkultur* Wien, 17.-20.09.1986 | 1986 | Vorträge | 1-142  +5 |
| 31a | *2. Sonderheft zum Internationalen Phänologie-Symposium an der Universität für Bodenkultur* Wien, 17.-20.09.1986 Ergebnisse aus den Internationalen Phänologischen Gärten (IPG) Europas, Mittel 1973-1982. | 1987 | Schnelle, F.  Schnelle, F. | 1-9  10-15 |
| 32 | *Aufbau, Nutzen und Konsequenzen einer IPG-Datenbank* Bemerkungen zu den phänologischen Beobachtungen im Jahr 1985. | 1987 | Freitag, E. Polte, C. | 1-12  13-31 |
| 33 | *IPG in der Bundesrepublik Deutschland*  Phänologische Beobachtungen im Nationalpark Bayerischer Wald. Bemerkungen zu den phänologischen Beobachtungen im Jahr 1986. | 1988 | Haug, M. Freitag, E.; Meier, D. | 1-20 21-39 |
|  | **Herausgeber: Prof. Dr. H. Lieth und Dipl. Met. H. Scharrer** |  |  |  |
| 34 | *Nachruf Dr. Fritz Schnelle* Bemerkungen zu den phänologischen Beobachtungen im Jahr 1987 und 1988. Jahresunterschiede des Frühlingsbeginns in Nord-, Mittel- und Südeuropa. | 1991 | Scharrer, H. Polte-Rudolf, C. Schnelle, F. | 1-9 10-34 35-40 |
| 35 | *Phänologie und Fernerkundung* Untersuchung der Saisonalität der Vegetation anhand eines Vergleiches von phänologischen Daten mit Daten aus der Fernerkundung. Bemerkungen zu den phänologischen Beobachtungen 1989 und 1990. | 1991 | Lohmann, M.  Polte-Rudolf, C. | 1-19  20-47 |
| 36 | *Austriebsverhalten von Fichtenklonen*  Austriebsverhalten von Fichtenklonen *(Picea abies* L.) in unterschiedlichen geographischen Gebieten. Was geschieht mit den IPG? Bemerkungen zu den phänologischen Beobachtungen im Jahr 1991. | 1992 | Hanhart-Rosch, R.; Kleinschmidt, J. Scharrer, H.  Polte-Rudolf, C. | 1-7  8-17 18-35 |
| 37 | *Mediterranean Bush Phenology as a function of Temperature* Aktivitäten und Hinweise in Sachen IPG. Bemerkungen zu den phänologischen Beobachtungen im Jahre 1992. | 1993 | Bagella, S.; Deidda, P.  Scharrer, H. Polte-Rudolf, C. | 1-19 20-25 26-49 |
| 38 | *Sonderheft IPG Datenbank*  Die IPG-Datenbank im Deutschen Wetterdienst.  Der Nutzen der IPG-Datenbank. | 1993 | Polte-Rudolf, C.  Scharrer, H. | 1-28 29-37 |
| 39 | *Untersuchungen zur Problematik der Mittelwertbildung bei phänologischen Datenreihen* Zukunft der Internationalen Phänologischen Gärten.  Bemerkungen zu den Phänologischen Beobachtungen im Jahre 1993. | 1994 | Gornik, W.  Scharrer, H. Polte-Rudolf, C. | 1-13 14-17 18-51 |
| 40 | *Humboldt-Universität zu Berlin übernimmt IPG!*  Klimaänderung im Spiegel phänologischer Zeitreihen. Weitere Untersuchungen zur Problematik der Mittelwertbildung bei phänologischen Datenreihen.  Bemerkungen zu den phänologischen Beobachtungen im Jahr 1994. | 1995 | Scharrer, H. Rötzer, T.; Sachweh, M. Gornik, W.  Polte-Rudolf, C. | 1-7 8-22 23-27 28-59 |
|  | **Herausgeber: Dr. F.-M. Chmielewski** |  |  |  |
| 41 | *Phenological Observations 1995*  Ergebnisse der Internationalen Phänologischen Gärten 1959-1993.  Internationalen Phänologischen Gärten 1959-1997. | 1998 | Menzel, A. Chmielewski, F.-M. | 1-9 10-35 |
| 42 | *Phenological Observations from the Years 1996 and 1997.* Relationships between Grapevine Phenology, Composition, and Quality for Bordeaux, France. Internationale Phänologische Gärten 1959-1997 und 1996/1997. | 1999 | Gregory V.J. Chmielewski, F.-M. | 1-7 8-46 |
| 43 | not available in the archive |  |  |  |
| 44 | *Results of the Research-Project: Climate Variability and Phenology in Central Europe*  Zum 100. Geburtstag von Dr. Fritz Schnelle.  Using the IPG observations to test for model estimates stability across environments.  Klimavariabilität und Phänologie in Europa. Der neue IPG in Prag-Doksany.  Tabellen und Abbildungen; Phänologische Beobachtungen von 1998 und 1999. Beobachtungsanleitung der Internationalen Phänologischen Gärten. | 2001 | Chmielewski, F.-M. Chuine, I.  Chmielewski, F.-M.; Rötzer, T. Chmielewski, F.-M.  Rötzer, T. | 1-5 6-8 9-16 17-19 20-65 58-67 |
| 45 | *Climate Change Indicators for Ireland; Phenological Observations of 2000*  BBCH-code of phenophases from the IPG-programme.  Trees as climate change indicators for Ireland.  Phenological studies of *Sambucus nigra.* Tabellen und Abbildungen; Phänologische Beobachtungen von 2002 Climate changes and trends in phenology of fruit trees and field crops in Germany, 1961-2000. | 2002 | Bruns, E.  Donelly, A.  Atkinson, M.D. Chmielewski, F.-M.  Chmielewski, F.-M.; Müller, A; Bruns, E. | 1-6 7-19 20-26 27-49  50-58 |
| 46/ 47 | *The Growing Network: 11 new IPGs in 2003 and 2004*  Mögliche Auswirkungen klimatischer Veränderungen auf die Vegetationsentwicklung in Sachsen Tabellen und Abbildungen; Phänologische Beobachtungen von 2001 und 2002. | 2003/  2004 | Chmielewski, F.-M.; Müller, A., Küchler, W. | 7-9  10-52 |
| 48 | *Maximum number IPG stations since 1959* IPG-Online: Working with the new IPG Online Interface. Spring phenological events in Slovenia related to air temperatures.  A 40-year study period of tree phenology at Tharandt IPG.  Tabellen und Abbildungen; Phänologische Beobachtungen von 2003. | 2005 | Doberev, G. Ĉrepinšek, Z.  Köstner, B.; Niemand, C.; Prasse, H.  Chmielewski, F.-M. | 3-8  9-18  19-23  24-47 |
